# Editorial: Physical and Cognitive Frailty in the Elderly: An Interdisciplinary Approach

**DOI:** 10.3389/fpsyg.2021.698819

**Published:** 2021-06-02

**Authors:** Martina Amanzio, Sara Palermo

**Affiliations:** ^1^Department of Psychology, University of Turin, Turin, Italy; ^2^Neuroradiology Unit, Neurological Institute C. Besta IRCCS Foundation, Milan, Italy; ^3^European Innovation Partnership on Active and Healthy Ageing, Brussels, Belgium

**Keywords:** physical and cognitive frailty, aging, mild cognitive impairment, major neurocognitive disorders, assessment, interventions

## Introduction

The scientific contributions published in the Research Topic emphasize frailty as a complex and heterogeneous clinical state which is described as the loss of harmonious interactions among various dimensions, such as biological, functional, psychological, cognitive, and social domains leading to homeostatic instability.

Data and inferences derived from different models currently in use to study frailty in aging individuals are presented: (1) the *biomedical approach*—represented by the Fried's frailty phenotype model (Fried et al., 2001)—which highlights a reduction in the ability to preserve homeostasis and respond to environmental changes appropriately; (2) the *bio-psycho-social model* (Gobbens et al., 2010), which defines the importance of a multidimensional approach to frailty, considering it no longer just a pathophysiological syndrome, but assessing its neuropsychological and social implications, especially considering different frailty states in individuals with neurocognitive disorders. Indeed, a fundamental question that needs to be clarified regards the relationship between neuropsychological dysfunctions and physical frailty. Up to now, this relationship is described as a “feedback loop relationship.” However, there is still a lack of knowledge about the mutual relationship between neuropsychological dysfunctions and physical frailty when considering the continuum from physiological aging to major neurocognitive disorders.

Three different reviews emphasized the different approaches by outlining:

1. A possible association between frailty and executive dysfunction in mild and major neurocognitive disorders due to Alzheimer's and Parkinson's diseases (Bartoli et al., 2020);
2. The critical determinants of frailty syndrome from a multidimensional perspective in cardiological conditions (Wleklik et al.);
3. A possible intervention model based on an integrated approach to proactively manage community-dwelling older people with suspected frailty (Lauretani et al.).

Indeed, the literature emphasizes the importance of developing programs that reverse the course of frailty while reducing the health, psychological, social, and economic costs of its negative consequences.

## The Biomedical Approach

Two original research are contextualized within the Fried's frailty phenotype model, which, based allows individuals to be classified as robust, pre-frailty or frailty on the number of lacking factors out of the five main components (weakness, sluggishness, involuntary weight loss, exhaustion, and low physical activity).

The first article investigates the specific contribution of each of the Fried's frailty components in a sample of 142 oldest old community-dwelling people. A multiple correspondence analysis has made it possible to identify two main facets of frailty: one related with physical components and the second related with intrinsic conditions (Alves et al.).

The second article investigates whether the frailty phenotype has a different association with hearing loss (HL) and tinnitus in 429 community-dwelling older adults. Authors found that frailty phenotypes showed divergent association with HL and tinnitus (Ruan, Chen, Zhang, Ruan et al.).

## The Multidimensional Approach—Valuing Cognitive Frailty

Cognitive decline and impaired global cognition have been mostly linked to frailty in the elderly. *Cognitive frailty* refers to the co-occurrence of mild cognitive impairment and physical frailty in the absence of a diagnosed major neurocognitive disorder (MND).

Two original articles suggest the importance of:

1. *screening cognitive frailty* with short cognitive screening instruments, analyzing their diagnostic accuracy in a Chinese population of 95 outpatients in rehabilitation clinics and suffering from subjective cognitive disorder, mild cognitive impairment and major neurocognitive disorder (Xu et al.).
2. *applying objective assessments* for the diagnosis of cognitive frailty subtypes, analyzing it in 335 community-dwelling older adults suffering from subjective cognitive decline and mild cognitive impairment compared to 144 robust elderly with normal cognition (Ruan, Chen, Zhang, Zhang et al.)

The general concept of looking more deeply into the cognitive correlates of frailty is interesting and may shed light on a more comprehensive model of cognitive frailty. This aspect becomes even more pervasive in the case of MND. This aspect is addressed by an original research which aims to identify predictors of those severe conditions in a sample of 250 adults, 30.4% of whom were classified as having probable major neurocognitive disorder (Sousa et al.). Authors found that advanced age, school education, physical activity, and hand strength are major predictors of MND.

These findings support the findings of previous literature, which has shown that pre-frailty already impact on executive-metacognitive functions and behavior in minor and major neurocognitive disorders (Amanzio et al., 2017). Moreover, a correlation between a specific pattern of co-occurring gray matter atrophy and hypometabolism with pre-frailty has been found in behavioral variant frontotemporal dementia (Amanzio et al., 2021), paving the way for this type of investigation in other types of neurodegeneration as well.

## The Multidimensional Approach—Valuing Physical Activity

Inadequate physical activity is associated to higher probability for frailty in the elderly (da Silva et al., 2019). Four original articles address this matter.

A first study aims to determine whether grip strength loss is a convincing predictor of impairment in cognitive performance and social functioning in 30 patients with type-2 diabetes mellitus and 107 subjects with severe mental illnesses (35 major depressive disorder, 42 bipolar disorder, 30 schizophrenia) in a 1-year longitudinal study (Aliño-Dies et al.). Authors concluded that interventions aimed to improve the overall physical conditions of patients who have poor grip strength could have beneficial effects on global cognition and social functioning.

A second original research sought to investigate the possible long-term association between activity, physical and cognitive functions in 10,240 middle-aged and elderly people through a multivariate latent growth modeling (Bae). Moreover, it was verified whether there is a long-term mediating effect of physical activity on the relation between social activity and cognitive function. The author suggested that social activity had a positive impact on cognitive function and negative impact on physical function decline. Furthermore, a decline in physical activity affected cognitive function through the indirect action of social activity.

A cross-sectional study aims to explore the interactive relation between physical frailty and psychological well-being on 358 older Portuguese women, finding that emotional well-being and global cognitive performance are strongly associated with physical frailty (Furtado, Caldo et al.). Authors concluded that the implementation of active lifestyle interventions to enhance positive psychological outcomes could help in the physical and mental health care of institutionalized elderly patients.

Finally, a clinical trial verified the potential beneficial impact of a 14-week combined chair-based exercise program (CEP) on immune/anti-microbial functions, salivary steroid hormones, functional fitness, and mental well-being in 47 pre-frail older women. Authors concluded that CEP is effective in improving performance in static balance and gait speed, immune and anti-microbial response, and happiness, while decreasing feelings of stress (Furtado, Letieri et al.).

An increasingly significant number of people find themselves in a condition of frailty, making this a hot topic. Just for an example, physical and cognitive frailty have proved to be more useful than ever in understanding the impact of the SARS-CoV-2 pandemic on the elderly population (Maltese et al., 2020; Bartoli et al.) and in guiding clinical vaccine trials principles (Palermo, 2020).

Research and attention to the issues proposed in this Research Topic will be increasingly necessary to ensure effective public health and welfare policies.

## Author Contributions

All authors listed have made a substantial, direct and intellectual contribution to the work, and approved it for publication.

## Conflict of Interest

The authors declare that the research was conducted in the absence of any commercial or financial relationships that could be construed as a potential conflict of interest.
